# SCID MOUSE MODEL OF PSORIASIS: A UNIQUE TOOL FOR DRUG DEVELOPMENT OF AUTOREACTIVE T-CELL AND TH-17 CELL-MEDIATED AUTOIMMUNE DISEASES

**DOI:** 10.4103/0019-5154.62752

**Published:** 2010

**Authors:** Smriti K Raychaudhuri, Siba P Raychaudhuri

**Affiliations:** *From the Division of Rheumatology, Allergy and Clinical Immunology, University of California Davis, School of Medicine and VA Sacramento Medical Center, CA 95618, USA.*

**Keywords:** *Novel therapy*, *psoriasis*, *psoriatic arthritis*, *SCID mouse*, *Th17 cell*

## Abstract

In both skin and synovial tissues of psoriatic arthritis (PsA) patients, there are prominent lymphocytic infiltrates localized to the dermal papillae in the skin and the sublining layer stroma in the joint. T-cells, with a predominance of CD4+ lymphocytes, are the most significant lymphocytes in the tissues; in contrast, this ratio is reversed in the epidermis, synovial fluid compartment, and at the enthesis, where CD8+ T-cells are more common. This differential tropism of CD8+ T-cell suggests that the CD8+ T-cells may be driving the immune response in the joint and skin. This is supported by an association with MHC class I. The cytokine network in the psoriatic skin and synovium is dominated by monocyte and T-cell-derived cytokines: IL-1β, IL-2, IL-10, IFN-γ, and TNF-α. In PsA synovium, higher levels of IFN-γ, IL-2, and IL-10 have been detected than in psoriatic skin. An analysis of T-cell receptor beta-chain variable (TCRβV) gene repertoires revealed common expansions in both skin and synovial inflammatory sites, suggesting an important role for cognate T-cell responses in the pathogenesis of PsA and that the inciting antigen may be identical or homologous between the afflicted skin and synovium. Traditionally, T-cells have been classified as T helper 1 (Th1) or Th2 cells by production of defining cytokines, IFN-γ and IL-4, respectively. Recently, a new type of T-cell, Th17, has been linked to autoimmune inflammation. T-helper 17 (Th17) cells are a unique effector CD4+ T-cell subset characterized by the production of interleukin (IL)-17. Murine diseases that were previously considered to be pure Th1-mediated responses have been shown to contain mixed populations of Th1 and Th17 cells. Also, in humans, a critical immunoregulatory role of Th-17 cells in infectious and autoimmune diseases has been identified. It has been postulated that IL-17 may be important in psoriasis. Our initial observations demonstrate that IL-17 and its receptor system are important for PsA also. In *in vivo* and *in vitro* studies we have demonstrated that IL-17/IL-17R are enriched in skin, synovial tissue, and synovial fluid of psoriatic arthritis patients and Th17 cells are functionally significant in the pathogenesis of psoriasis and psoriatic arthritis. Here we will share our experience of the SCID mouse model of psoriasis in respect to its use in investigating psoriatic diseases and development of immune-based drugs for psoriasis, psoriatic arthritis, and other autoimmune diseases.

## Introduction

Psoriasis is a multifactorial chronic inflammatory disease.[1] The exact cause of psoriasis and its associated psoriatic arthritis (PsA) has yet to be identified. Genetic, immunologic, and environmental factors contribute to its pathogenesis.[1–3] PsA is a systemic inflammatory disease involving mainly the skin and joints. Psoriasis and PsA have similarities in HLA phenotyping, cell trafficking mechanisms, the nature of T-cell phenotypes, cytokine profiles, and angiogenesis. It is reasonable to postulate that the skin and joint involvement share common pathophysiologic processes. However, there are also several differences in genetic, clinical presentation, therapeutic response, and pathophysiologic events in patients with psoriasis and PsA. The severity of the skin disease and arthritis often does not correlate. Patients with severe PsA may not have extensive skin lesions; patients with severe psoriasis may not have arthritis. About 40% of patients with psoriasis or PsA have a family history of these disorders in first-degree relatives.[1,2] The HLA antigens B13, B17, B39, and Cw6 occur with increased frequency in both psoriasis and PsA.[2,3] In PsA, additional associations have been found with HLA-B38, HLA-B39, HLA-DR4, and with HLA-B27 in patients with predominant spinal disease.[2]

Despite intense research work on psoriasis, the etiology is unknown and its treatment remains palliative. The lack of an animal model has been a major hurdle for the investigation of the cause and cure of psoriasis. Since there are no naturally occurring diseases in animals that exhibit all the phenotypic and immunological features of psoriasis, several approaches have been utilized, which include studies on mutant strains of mice, development of transgenic mice, and xenotransplantation models.[4–6]

## Xenotransplantation Model

Animal models based on transgenic technology have been used extensively to study the pathogenesis of various diseases, including psoriasis. As these models are created by manipulating a single gene, usually they do not represent the phenotype of these complex inflammatory diseases. Psoriasis especially, being a polygenic disease, cannot be truly reproduced in a model system by the manipulation of a single gene. In that respect xenogenic transplantation models allow investigation of a disease process in a microenvironment resembling its natural milieu. Genetically, immunodeficient mice have been used in a xenograft model as they fail to reject skin grafts. Among the xenogenic transplantation models, nude mice and SCID mice have been used to elucidate the pathogenesis of psoriasis.[4–6] The thymus is not functional in the nude mouse. Thus, the nude mouse lacks the T-cell arm of the immune system. Transplanted psoriatic plaques in nude mice develop certain histological changes that are not typical of psoriasis, such as the absence of parakeratosis and the presence of a granular layer. In addition, circulating immunoglobulins impair immunohistochemical evaluation. As SCID mice lack both B and T-cells they are better recipients of grafts than nude mice.

## SCID Mouse-Psoriasis Skin Xenograft Model

**Figure 1:**
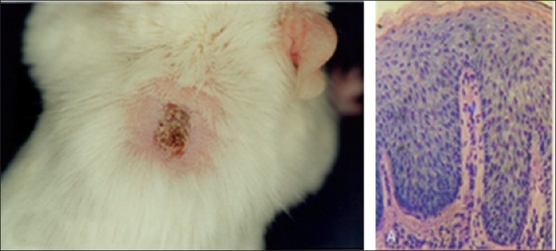
Clinical and histological features typical of psoriasis are maintained in a transplanted plaque of 12 week duration

To establish this model, we collect 0.5 mm thick keratome shave biopsy samples (2.5 × 2.5 cm) from patients with chronic plaque psoriasis. 3-4 grafts approximately of 1 × 1 cm are grafted onto BALB/cByJSmn-Prkdc^scid^/J SCID mice. Histological features in transplanted skin have been reported to be maintained for several months. In transplanted grafts of psoriatic tissue, we have noted that the clinical, histological, and immunological features of psoriasis could be maintained for a duration of 16-20 weeks. Figure 1 demonstrates clinical and histological features typical of psoriasis from a transplanted plaque of 12 week duration.

The inflammatory reaction in psoriasis is uniquely characterized by increased expression of adhesion molecules on the endothelial cells (E-selectin, ICAM, VCAM)[7–11] proliferation of vessels (angiogenesis) with upregulation of endothelial cell-stimulating angiogenesis factor (ESAF) and VEGF[12,13] infiltrates containing activated T-cells (CD4, CD8, NK cells)[14–16] neutrophils (Munro's and Kogoji microabscess), mast cells[17,18] marked upregulation of chemokines such as IL-8, RANTES, fractalkine[19–22] and neuropeptides.[23–26] As medicines can be delivered at the site of the lesion, and a therapeutic response can be determined mostly by clinical improvement, many investigators consider psoriasis an ideal disease model for evaluating the efficacy of new-generation anti-inflammatory drugs. Thus, the SCID mouse model is of immense help to target any of these molecules and develop novel drugs for psoriasis and other autoimmune diseases.

Following molecules are identified to be maintained for the lifetime in the transplanted plaques on SCID mice: p38 MAPK, STAT3, ICAM, CXCR3, Fractalkine, IL-8, CD3, CD4, CD8, HLA-DR, CD40, OX-40R, CD80, CD86, K16, Ki67, substance P, NGF, NGF-R. Several co-stimulatory molecules have a critical role for T-cell activation. Among these a principal signal is delivered by the engagement of CD28 on T-cells with CD80 (B7-1) and CD86 (B7-2) on APCs. TCR-mediated signal cascades are intact in the transplanted grafts; in a recent study, we identified ZAP70 in its phosphorylated state. Langerhans cells, dermal dendrocytes, and the activated T-cells express CD80 and CD86 the ligands of the co-stimulatory molecules.

Upregulation of CD80/CD86 in psoriatic lesions suggests a critical role for the CD28/B7 co-stimulatory system in the pathogenesis of psoriasis. Thus, inhibition of the CD28 and CD80/CD86 interaction is expected to restrict the inflammatory processes of psoriasis. Antagonism of co-stimulatory molecules has provided a new dimension for treatment of the autoimmune diseases.[5] To have a proof of the concept that psoriasis SCID mouse model can be used to evaluate therapeutic efficacy of pharmacologic agents targeting the T-cell co-stimulatory system, we designed a double-blinded, placebo controlled study using the SCID-psoriasis xenografts.[5] The transplanted psoriatic plaques on the SCID mice (*n* = 12) were treated with CTLA4IgG (10 mg/kg/week), a natural inhibitor of CD28/B7 co-stimulatory signals. Cyclosporine (4mg/kg) treatment was used as a positive control group (*n* = 6) and untreated plaques (*n* = 12) were negative controls. CTLA4IgG-treated plaques significantly improved following 4 weeks of therapy. The length of the rete pegs changed from 308.57±98.72 mm to 164.64±46.78 mm (*P*<0.01, Student's *t*-test). A similar improvement of psoriasis was observed in the cyclosporine group, whereas the untreated plaques did not show any improvement. Significant reduction of lymphomononuclear cells, HLA-DR, and ZAP70 expression was observed in the plaques treated with CTLA4IgG and cyclosporine. In addition to systemic therapy, this model can be used to develop novel topical pharmacologic agents. Various established topical preparations for psoriasis such as steroid and retinoid creams are highly therapeutically effective in this model. Manipulation of the CD28-B7 co-stimulatory system by an antisense CD28 nucleotide cream induced significant histological improvement with marked reduction of the activated T-cells. This model is of immense help to develop immune-based therapy for T-cell-mediated autoimmune diseases by targeting regulatory molecules of the co-stimulatory system and specific phosphokinases associated with T-cell activation.

### Th17 cells and psoriatic disease

**Figure 2:**
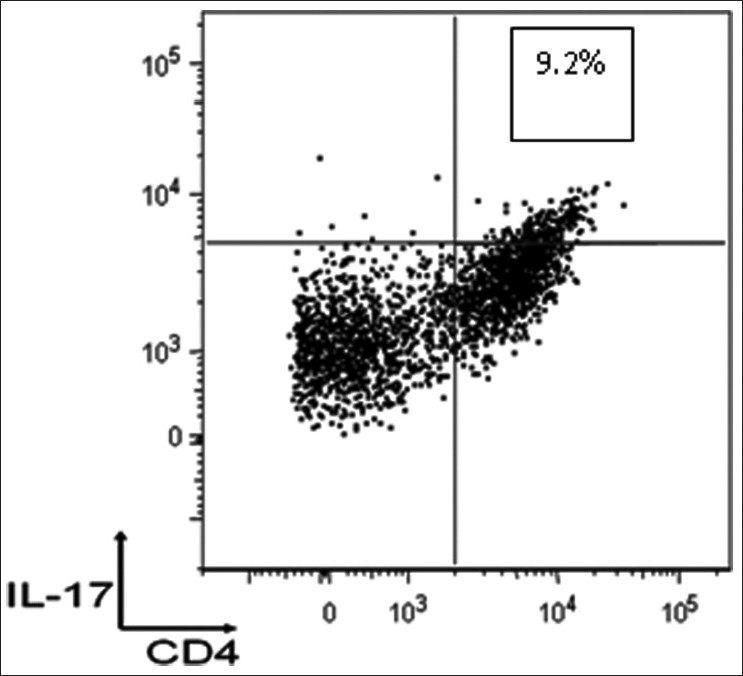
Synovial fluid mononuclear cells from a psoriatic arthritis patient stained for IL-17 expression by multiparameter FACS analysis. The figure shows that IL-17 expression is restricted to CD4+ lymphocytes and 9.2% of CD4+ lymphocytes express IL-17

**Figure 3:**
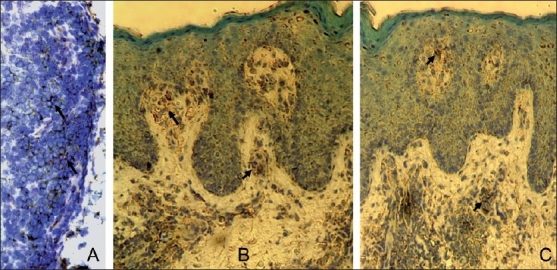
Immunoperoxidase staining performed with an IL-17 antibody in psoriatic arthritis synovial tissue (Fig 3 A), psoriasis plaque (Fig 3B), transplanted psoriasis plaque on SCID mouse (Fig 3 C). Significant number of Th-17 cells were identified in psoriatic synovial tissue and psoriatic plaques (Reference number 35). Dark brown staining indicated by arrows represents IL17+ cells

IL-17 is produced by a unique subset of helper T-cells, Th-17 cell.[27] The binding of IL-17 to an IL-17 receptor expressed on epithelial, endothelial, or fibroblastic stromal cells induces robust effects on stromal cells in many tissues. Through the ubiquitously expressed IL-17 receptor, IL-17 induces the secretion of proinflammatory cytokines,[28] recruits neutrophils/monocytes, and triggers an immune-mediated inflammatory reaction.[29,30] Recent animal studies revealed critical roles of interleukin (IL)-17 in the development of autoimmune diseases.[31,32] Detailed characteristics and the prevalence of Th-17 cells in human autoimmune diseases like in psoriasis and inflammatory arthritis are in the developing phase.[33,34] In order to extend our knowledge about the role of IL-17 in the pathogenesis of psoriatic disease, we carried out extensive *in vivo*/*in vitro* studies.[35] We have identified the T_h_17 cells in psoriatic arthritis and determined their phenotypes and functional significance [Figure 2]. Compared to OA patients, the frequency of Th-17 cells increased in synovial fluid of PsA patients by 10-fold (*P*<.001). Th-17 cells were significantly higher in psoriatic synovial tissue and psoriatic plaques compared to the controls [Figure 3]. In the psoriatic lesion, Th-17 cells were predominantly localized in the papillary dermis. IL-17 has many proinflammatory effects in a wide variety of cells including keratinocytes, macrophages, and endothelial cells. Downstream effects of IL-17 include production of IL-1, IL-6, IL-8, TNF-α, G-CSF, and GM-CSF, as well as anti microbial peptides. Identification of Th-17 cells in skin and synovium of psoriatic tissue suggests their possible pathologic role in the psoriatic disease process. The hypothesis that Th-17 cells may play a pivotal role in psoriatic disease needs further investigations. To determine the role of T_h_17 cells in maintenance of psoriatic lesion we studied the T_h_17 cells in the SCID mouse-psoriasis skin xenografts. IL-17+T-cells could be identified in the transplanted psoriasis lesions on SCID mice. In Figure 3 (panel C) in a biopsy collected at 12th week of transplantation demonstrates large numbers of IL17+cells in the upper dermis. Th-17 cells may offer a rational therapeutic target for psoriasis and psoriatic arthritis. Currently, using the SCID mouse model of psoriasis, we are exploring to develop treatment of autoimmune diseases by targeting Th17 cells with IL-17 and IL-17R antibody.
